# Genetic Polymorphisms in Long Noncoding RNA H19 Are Associated With Susceptibility to Breast Cancer in Chinese Population

**DOI:** 10.1097/MD.0000000000002771

**Published:** 2016-02-18

**Authors:** Zongjiang Xia, Rui Yan, Fujiao Duan, Chunhua Song, Peng Wang, Kaijuan Wang

**Affiliations:** From the Department of Surgery Medicine, Division of Thoracic Surgery, The First Affiliated Hospital, Zhengzhou University (ZX); Department of Epidemiology and Statistics, College of Public Health, Zhengzhou University (RY, CS, PW, KW); Henan Key Laboratory of Tumor Epidemiology (RY, CS, PW, KW); and Department of Hospital Infection Management, Affiliated Cancer Hospital of Zhengzhou University (FD), Zhengzhou, China.

## Abstract

H19, a maternally expressed imprinted gene transcribing a long noncoding RNA, has previously been reported to be involved in tumorigenesis and cancer progression. However, the association between the H19 polymorphisms and breast cancer (BC) susceptibility has remained elusive. The aim of this study was to evaluate the associations between 2 H19 haplotype tagging SNPs (rs3741219 T>C, rs217727 C>T) and the risk of breast cancer.

Our study comprised 464 BC patients and 467 cancer-free controls in China. rs3741219 and rs217727 were genotyped with polymerase chain reaction-restriction fragment-length polymorphism (PCR-RFLP) and created restriction site PCR (CRS-RFLP) assays, respectively. False-positive report probability (FPRP) was calculated to test the false-positive association.

On performing univariate analysis, no significant association between H19 polymorphisms (rs3741219 and rs217727) and BC was observed. However, in further stratified analyses, CT+TT genotypes of rs217727 had a significantly lower risk of breast cancer among women with number of pregnancy >2 (OR = 0.79; 95% CI = 0.55–0.97). CT genotype of rs217727 was associated with ER positivity (OR = 2.19; 95 % CI = 1.07–4.45) and HER-2 positivity (OR = 1.34; 95 % CI = 1.05–2.12). It was proved that our results were less likely to be false positives according to false-positive report probability calculation.

Our findings extend available data on the association of H19 polymorphisms and BC susceptibility. Further validation in large population or cohort studies is needed.

## INTRODUCTION

Breast cancer is the most frequently diagnosed malignant tumor and the first leading cause of cancer death among women.^1–3^ The development of BC is a complex and multifactorial process, and numerous epidemiological studies have identified several risk factors for BC, involving multiple reproductive factors and susceptibility genes.^4,5^ Previous genomics researches have provided evidence to support that single nucleotide polymorphisms (SNPs) in several genes were associated with the risk of BC^6,7^ and may contribute to the carcinogenesis of BC.

Long noncoding RNAs (lncRNAs) are defined as transcribed RNA molecules that are >200 nucleotides, lacking open reading frame, and having no obvious protein-coding capacity.^8,9^ Several lncRNAs have been reported to be associated with cancers,^10,11^ including BC. The handful of aberrantly expressed lncRNAs in different cancers suggests that lncRNAs contribute to carcinogenesis, and deliver functions in controlling cell cycle progression, apoptosis, invasion, and migration.^12^ Single nucleotide polymorphisms (SNPs) have been confirmed to have profound effects on gene expression and function, and participate in carcinogenesis. Recently, studies on the effects of SNPs have extended to functional lncRNAs. For example, HOTAIR has been widely identified to participate in tumor pathogenesis, acting as a promoter in colorectal cancer carcinogenesis.^13^ HOTAIR rs7958904 CC decreased the risk of colorectal cancer compared with GG genotype^14^ and HOTAIR rs920778 TT carriers had increased gastric cancer risk.^15^ Increased breast cancer risk was associated with the lincRNA-ENST00000515084 rs12325489 CC and CT genotype when compared with the rs12325489 TT genotype.^16^ rs11752942 AG+GG in the lincRNA-uc003opf.1 exon had a significantly reduced risk of esophageal squamous cell carcinoma.^17^

Another lncRNA that may play an important role in carcinogenesis is H19. LncRNA H19 is an imprinted gene, located on human chromosome 11p15.5, encodes a 2.3-kb long, capped, spliced, and polyadenylated noncoding RNA.^18^ H19 is abnormally expressed in several tumors, and it acts as either a tumor suppressor,^19,20^ or an oncogene.^21^ Increasing evidence suggests that H19 genetic variants play important roles in the development of cancers. For example, CT+TT rs2839698 in H19 was associated with significantly increased gastric cancer risk.^22^ A significantly decreased risk of bladder cancer was found for H19 rs2839698 TC carriers.^23^ However, according to our latest knowledge, no research has been executed to evaluate the H19 polymorphism and the risk of BC. On the basis of the above description, we hypothesized that functional SNPs in H19 might have association with the BC risk. We genotyped 2 H19 haplotype tagging SNPs in a population-based case–control study comprising 464 BC patients and 467 age frequency matched controls from China. The associations between the H19 SNPs and breast cancer risk were investigated by molecular epidemiology.

## MATERIALS AND METHODS

### Study Population

There were 464 BC patients and 467 cancer-free controls in our study. All subjects were genetically unrelated ethnic Han Chinese women. Patients with newly pathologically confirmed breast cancer were recruited from the First Affiliated Hospital of Zhengzhou University and the Third Affiliated Hospital of Zhengzhou University between 2014 and 2015. We then selected 467 healthy participants, who are genetically unrelated with cases, free of any cancer and having no the history of chronic diseases, as our control group, from a database consisting of >20,000 subjects participated community-based chronic diseases program in Henan province during the same time period that the cases were collected. All healthy controls were frequency matched to the cancer patients on the basis of their age ( ± 2 years).

A structured questionnaire was used to retrieve reproductive information on the subjects, such as age, age of menarche, premenopausal or postmenopausal, age of menopause, number of pregnancy, number of abortion, breast-feeding history for born baby (yes, no), and family history of BC in first-degree relatives (yes, no). The questionnaires were administered by trained interviewers through face-to-face interviews, and informed consent was obtained from each study participant. Pathological data of BC patients, including of the estrogen receptor (ER), progesterone receptor (PR), and human epidermal growth factor receptor-2 (HER-2) status, were collected by immunohistochemistry (IHC) from the patients’ pathology reports. The study was approved by the ethical review committee of Zhengzhou University Committee for Medical and Health Research Ethics.

### DNA Extraction

Venous blood (5 mL) was collected into a test tube containing ethylene diamine tetra acetic acid (EDTA) from each BC patient and healthy women control. Genomic DNA was extracted from peripheral blood samples using the DNA Extraction Kit of TIANGEN BIOTECH (Beijing) according to the manufacturer's instructions. The extracted DNA was stored at −80°C until use.

### SNPs Selection and Genotyping

Haplotype-tagging SNPs in lncRNA H19 in the chromosomal region 11p15.5 were selected by using Haploview software 4.2, based on the Chinese Han population data of HapMap (HapMap Data Rel 28 Phase II + III, August 10, on NCBI B36 assembly, dbSNP b126). We searched tagging SNPs that captured all the known common SNPs located in the chromosome locus transcribed into H19 and its flanking region (2000 bp upstream and 2000 bp downstream, respectively). Finally, 2 tagging SNPs (rs3741219 T>C, rs217727 C>T) that located in the H19 were selected, with a pairwise correlation *r*^2^ >0.8. The genotyping of rs3741219 was determined by polymerase chain reaction-restriction fragment-length polymorphism (PCR-RFLP), whereas rs217727 was genotyped with created restriction site PCR (CRS-RFLP) assays.

The primers designed by Primer 6.0 software used for PCR amplification were 5′-CCCCCTGCGGCGGACGGTTGA -3′ (forward) and 5′-GGCGTAATGGAATGCTTGAA-3′ (reverse) for rs3741219, and 5′-ACTCAGGAATCGGCTCTGGAAGGTG-3′(forward) and 5′- GATGTGGTGGCTGGTGGTCAACGGT-3′(reverse) for rs217727 (Table 1). PCR primers were further verified by NCBI BLAST (http://blast. ncbi.nlm.nih.gov/Blast.cgi/) to assess the possibility of amplifiation of any nonspecific DNA sequences and synthesized commercially.

**TABLE 1:**
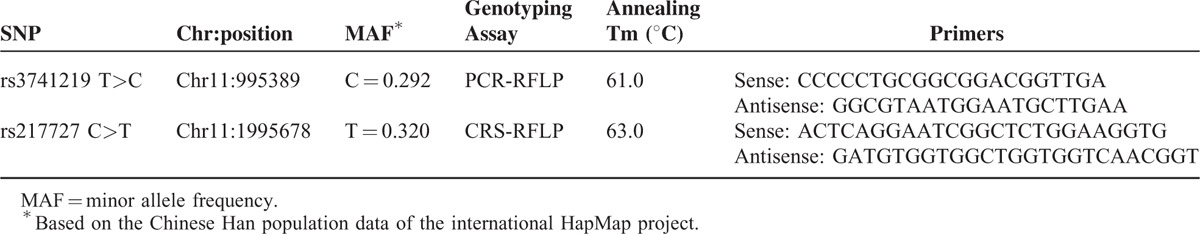
PCR Information of the 2 SNPs

For each sample, PCR amplification was performed in a final volume of 30 μL, which contained 15 μl 2 × Tap PCR MasterMix, 0.5 μL each primer (10 μM), 50 ng DNA, and 13 μL deionized water. Thermocycling conditions of PCR were as follows: initial denaturation at 95 °C for 5 min, 35 cycles of PCR consisting of denaturation at 94 °C for 30 s, optimal annealing temperature (61.0 °C for rs3741219, and 63.0 °C for rs217727) for 45 s and extension at 72 °C for 45 s, and final extension step of 72 °C for 5 min. In addition, the restriction enzyme HhaI and RsrII (Fermentas, Canada), selected by WATCUT website (http://watcut.uwaterloo.ca/watcut/watcut/template.php), were used for genotyping of rs3741219 and rs217727, respectively.

All analyses were performed without knowledge of the case or control status for quality control. Totally 10% of the study populations were randomly selected from both cases and controls to confirm the genotyping results by the direct sequencing (BGI Sequencing, Beijing). The results of confirmation were found to be 100% concordant.

### Analysis

Hardy–Weinberg equilibrium (HWE) was evaluated by a goodness-of-fit chi-squared (χ^2^)-test to compare the observed genotype frequencies with the expected ones among the cancer-free control subjects. The differences in the distributions of age, reproductive variables, as well as the SNPs genotype frequencies between BC cases and controls, were appraised by using Student's *t* test for continuous variables, whereas the chi-squared (χ^2^) test was used for categorical variables. The relationship between genetic variants and case–control status was examined using unconditional logistic regression analysis (Stepwise selection), with adjustments for age, age at menarche, status of menopausal, number of pregnancy, number of abortion, breast-feeding history for born baby, family history of BC in first-degree relatives. Odds ratio (OR) and its corresponding 95% confidence interval (95% CI) were calculated to estimate the strength of association between the H19 polymorphisms and risk of BC. Furthermore, the data were stratified by age and reproductive factors to evaluate the stratum variable-related ORs among various H19 SNPs. Haplotype analysis was conducted using the online SHEsis (http://analysis.bio-x.cn/myAnalysis.php). For all significant genetic effects observed in our study, the false-positive associations were calculated by FPRP with prior probabilities of 0.001, 0.01, 0.1, and 0.25. The OR was set at 1.5 under dominant genetic model, and a probability < 0.5 was considered as noteworthy. Statistical analysis was performed by using SPSS 16.0 software package (SPSS Inc., Chicago, IL). All tests were 2 sided and a *P* value < 0.05 was considered to be statistically significant.

## RESULTS

### Population Characteristics

The detailed demographic and reproductive characteristics of study subjects were illustrated in Table 2. The mean age was 48.44 ± 10.29 and 48.91 ± 9.99 years for BC cases and healthy controls, respectively. As expected, the mean age for 2 groups paired quite well. No significant differences between cases and control groups with respect to most of the baseline characteristic factors, including age, age at menarche and menopause, menstrual history, No. of abortion, breast-feeding, and family history. Only women with older age of menopause (>50) was found to be associated with significant increased BC risk (OR = 1.81; 95% CI = 1.07–3.06).

**TABLE 2:**
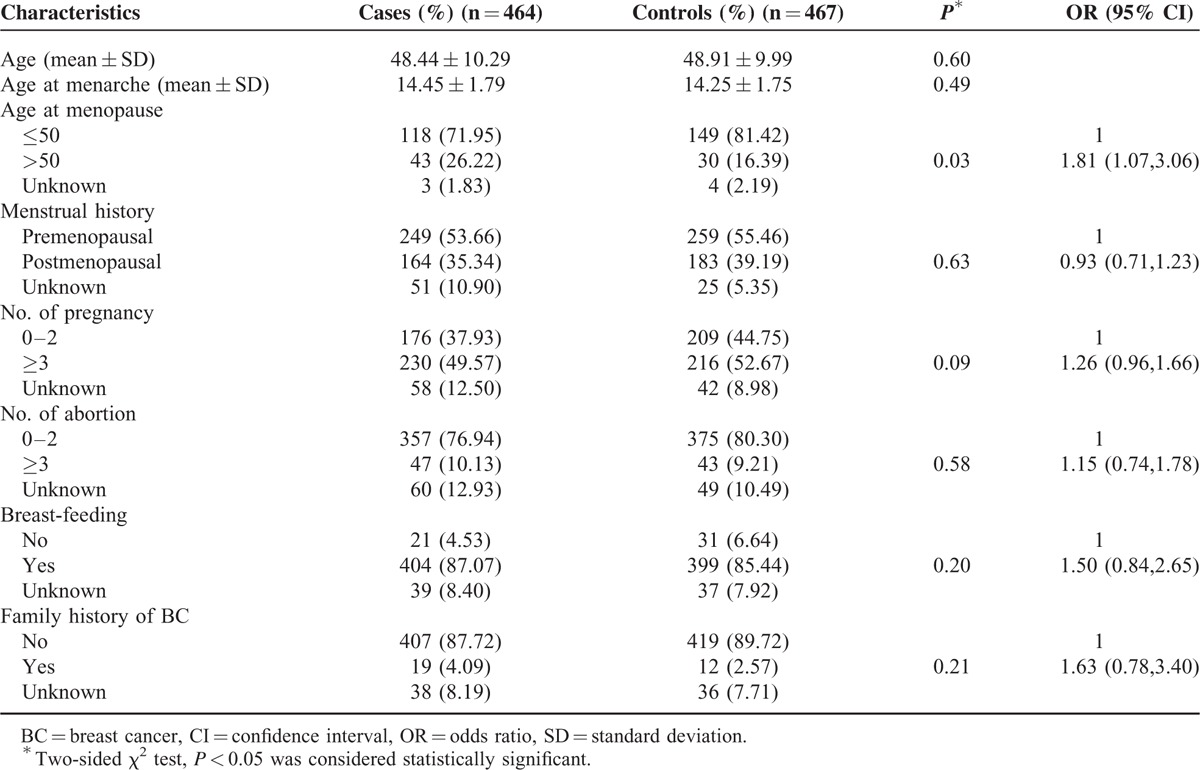
Characteristics of Breast Cancer Cases and Cancer-Free Controls

### H19 Genotypes and BC Risk

The genotype distribution of 2 tagSNPs and their associations with BC risk were shown in Table 3. No significant deviations from Hardy–Weinberg equilibrium for the 2 polymorphisms was found among the 467 healthy controls (*P* > 0.05). Genotyping results showed that only rs217727 CT was significantly associated with decreased BC risk (OR = 0.64; 95% CI = 0.47–0.87). However, the significant decreased BC risk was lost in multivariate analysis (OR = 0.88; 95% CI = 0.56–1.37), after adjusted for age, age at menopause, menopausal status, number of pregnancy and abortion, breast-feeding status and family history of BC in first-degree relatives. Neither the variant TC, CC, TC+CC genotypes nor C allele of rs3741219 had significant association with risk of BC.

**TABLE 3:**
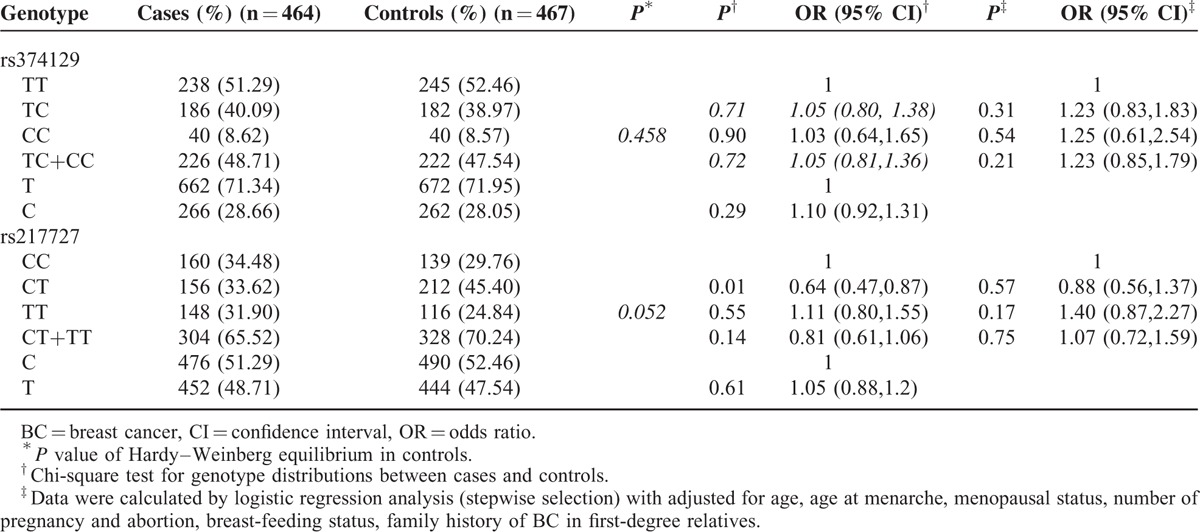
Genotype Among Cases and Controls and Their Association With BC Risk

### Haplotype Analyses

We further evaluated the combined effect of the 2 polymorphisms on the risk of BC by using the haplotype analysis. A total of 4 haplotypes were derived from the observed genotypes (Table 4), of which T_rs3741219_ T_rs217727_ was the most common haplotype in cases and controls. No significant association with BC risk was observed for these 4 haplotypes.

**TABLE 4:**
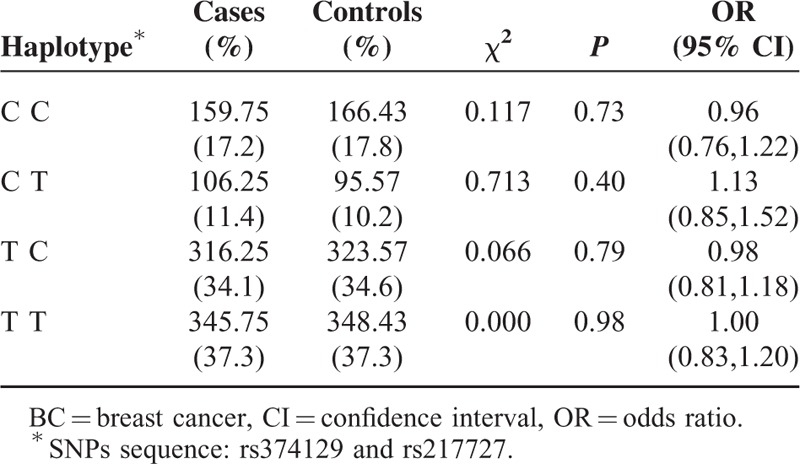
Haplotype Analysis of rs374129 and rs217727 Polymorphism Sites in H19

### Combined Effect of 2 SNPs

We then categorized the 2 SNPs into the number of combined variant alleles to further analyze their possible joint effect and potential locus–locus interaction on BC risk. As shown in Table 5, individuals with 1 mutation allele had 39% decreased BC risk when compared to individuals with 0 mutation allele (OR = 0.61; 95% CI = 0.40–0.91). No statistical risk for BC in other subgroups and no increased dose-dependent manner were observed on the combined effect of the 2 SNPs.

**TABLE 5:**
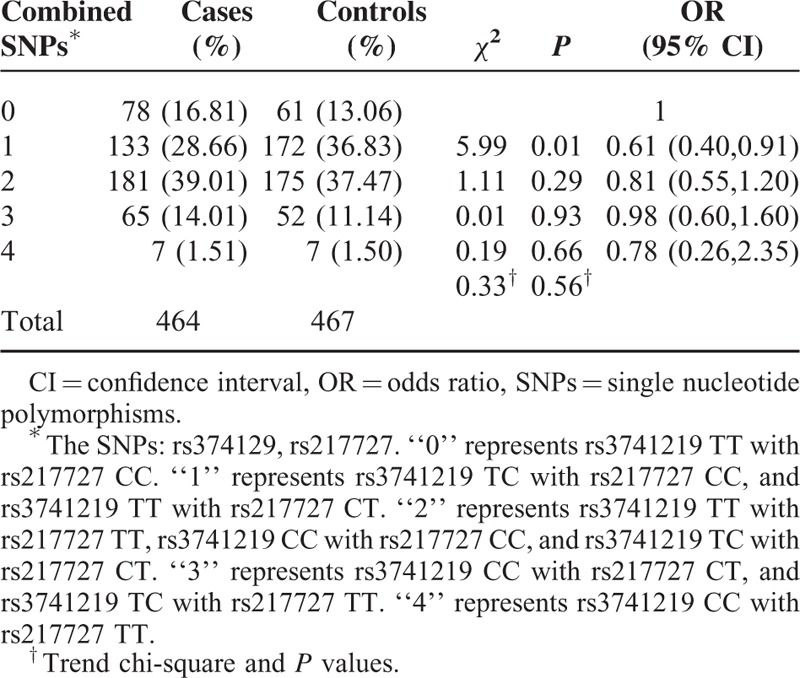
Combined Effect of the 2 SNPs on Breast Cancer

### Stratified Analysis of SNP Genotypes and BC Risk

Next, we performed a stratified analysis using logistic regression to evaluate the associations between H19 polymorphisms and BC risk. As indicated in Table 6, CT+TT genotypes of rs217727 had a significantly lower risk of breast cancer among women with no. of pregnancy >2 (OR = 0.79; 95% CI = 0.55–0.97).

**TABLE 6:**
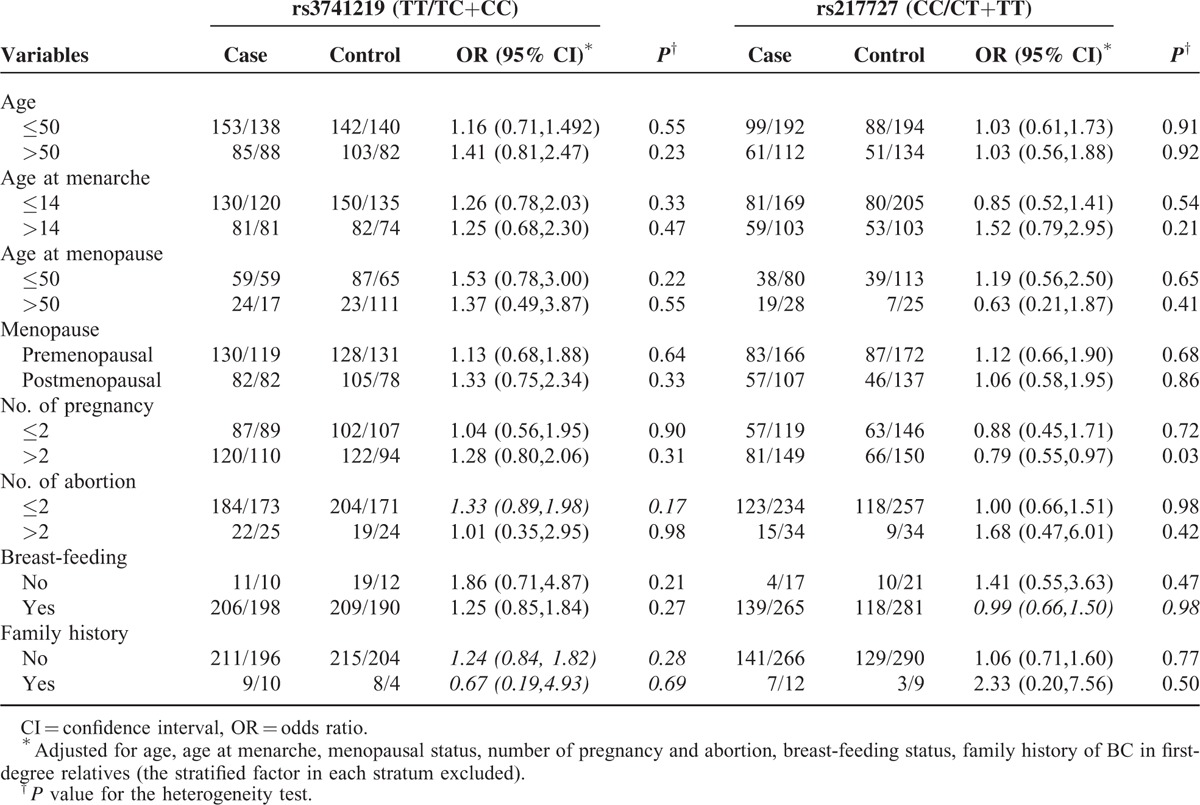
Stratification Analysis of the 2 SNPs Polymorphisms and BC Susceptibility

### Receptor Status and BC risk

We further demonstrated the association of rs3741219 and rs217727 polymorphism genotypes with the clinicopathological features in Table 7, including ER status, PR status and HER-2 status. In the case only analysis, compared with CC genotype of rs217727, CT genotype was associated with ER positivity (OR = 2.19; 95 % CI = 1.07–4.45) and HER-2 positivity (OR = 1.34; 95 % CI = 1.05–2.12). No significant association was found between rs3741219 variants and ER, PR, HER-2 status.

**TABLE 7:**
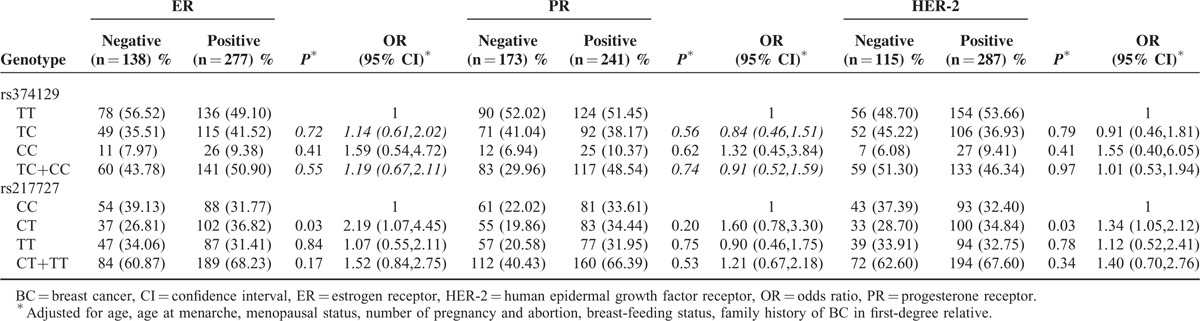
The Associations Between 2 SNPs and ER, PR and HER-2 Status of Breast Cancer Patients

### FPRP Calculation

We further calculated the FPRP values for all significant genetic effects observed in our study (Table 8). When we set the prior probability at 0.1, the association between rs217727 CT+TT genotypes and breast cancer for subjects with no. of pregnancy>2 was still significant (FPRP = 0.188). The positive association between individuals with 1 mutation allele and breast cancer was still noteworthy (FPRP = 0.295).

**TABLE 8:**
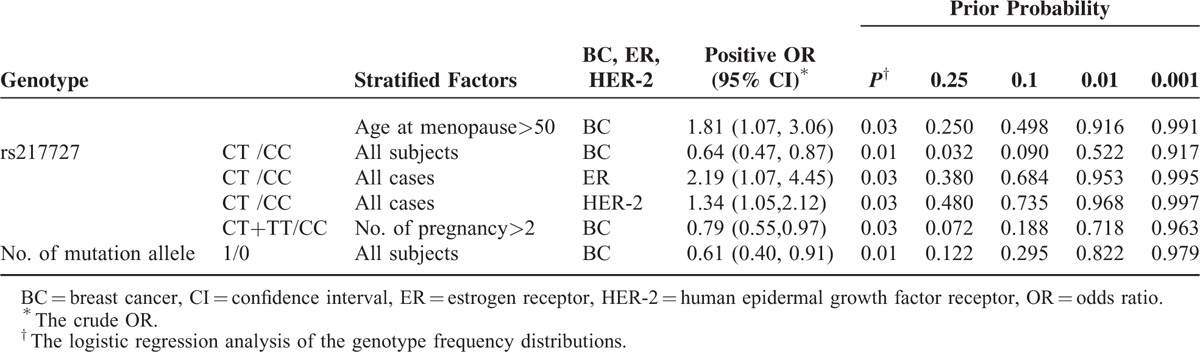
FPRP Values for Associations Between BC risk, ER, and Genotypes in Stratified Factors

## DISCUSSION

In the present molecular epidemiological study, we investigated the effects of 2 potentially functional polymorphisms (rs217727 and rs3741219) presented in H19 on BC susceptibility through a population-based case–control study in a Chinese Han population. To the best of our knowledge, this is the first study to examine the role of H19 polymorphism in BC tumorigenesis. No significant association between H19 polymorphisms (rs3741219 and rs217727) and BC risk was observed in our study. However, in further stratified analyses, CT+TT genotypes of rs217727 had a significantly lower risk of breast cancer among women with no. of pregnancy>2 (OR = 0.79; 95% CI = 0.55–0.97). CT genotype of rs217727 was significant associated with ER positivity (OR = 2.19; 95 % CI = 1.07–4.45) and HER-2 positivity (OR = 1.34; 95 % CI = 1.05–2.12). Our findings support the hypothesis that potentially functional SNPs of H19 may play a role in the etiology of BC and H19 genetic mutation may have interaction with ER and HER-2 during human breast tumorigenesis.

Previous studies have indicated that lncRNAs could deliver functions in controlling cell cycle progression, apoptosis, invasion, and migration. A genetic variation on lncRNA may change the lncRNA structure, affect the stability of the lncRNA, influence the level of lncRNA expression, and contribute to carcinogenesis. For example, a study by Wu et al, reported that when compared with the A allele, the rs11752942G allele could markedly attenuate the level of lincRNA-uc003opf.1 by binding micro-RNA-149, and affect esophageal squamous cell proliferation and tumor growth.^17^ Li et al founded that rs12325489 C to T base change could disrupt the binding site for miRNA-370, influencing lincRNA-ENST00000515084 transcriptional activity and affecting breast cancer cell proliferation.^16^ The H19 gene belongs to a highly conserved imprinted gene cluster that play important roles in embryonal development and growth control. The cluster also contains the nearby reciprocally imprinted gene for insulin-like growth factor 2 (IGF2).^24,25^ H19/IGF2 are closely linked and coordinately regulated by an intergenic control region (ICR) and a common enhancer region. In human, binding of the CTCF insulator protein to the nonmethylated ICR promotes H19 expression from the maternal allele, whereas IGF2 is expressed from the methylated allele. Recently, several H19 polymorphisms have been studied to be associated with diseases. For example, Gao et al found that rs217727 C to T variant was associated with increased coronary artery disease risk (CAD) (CT + TT vs CC: OR = 1.49; 95% CI = 1.21–1.83), whereas the rs2067051 G to A variant was associated with reduced CAD risk (GA + AA vs GG: OR = 0.77; 95% CI = 0.62–0.95).^26^ Another research shown that maternal H19 rs217727 TT genotype was associated with a higher birth weight.^27^ A population-based case–control study comprising 177 bladder cancer patients and 204 controls reported that rs2839698 TC genotype was statistical significantly associated with decreased risk of bladder cancer compared with TT carriers (OR = 0.60; 95% CI = 0.36–0.99). And a borderline significantly decreased risk of bladder cancer was found for rs217727 T allele carriers compared with C allele carriers (OR = 0.74; 95% CI = 0.51–1.06).^28^ However, a previous study by Yang et al evaluated the effects of 4 independent SNPs (H19 rs217727, rs2839698, rs3741216, and rs3741219) on gastric cancer risk in the Chinese Han population and suggested that the T allele of rs217727 was associated with higher risk of gastric cancer in both codominant (OR = 1.34; 95% CI = 1.03–1.73) and recessive (OR = 1.48; 95% CI = 1.04–2.10) inheritance genetic models^22^. Individuals with rs2839698 TT genotype had a significant increased risk of gastric cancer (OR = 1.64; 95% CI = 1.05–2.57) and carriers with rs2839698 T allele was significantly associated with higher H19 mRNA expression in cancer-free controls,^22^ suggesting a potential functional impact of H19 SNP on mRNA levels and the susceptibility to gastric cancer. In this population-based case–control study, we found a negative association between individuals with CT genetype of rs217727and BC susceptibility (OR = 0.64; 95% CI = 0.47–0.87). However, the significant association was lost in the logistic regression analysis (OR = 0.88; 95% CI = 0.56–1.37). Our results may require confirmation in larger prospective studies and different ethnicities in future.

By using the software program (http:// bioinfo.life.hust.edu.cn/lncRNASNP/), no miRNAs that combined with the C/T polymorphism rs217727 was found. However, we predicted that the T to C base change at rs3741219 T>C may create hsa-miR-1539 microRNA (miRNA) binding sites on H19. A single nucleotide polymorphism of lncRNA may change the lncRNA structure, affect the stability and influence the interaction of miRNA-lncRNA. Increasing evidence shows that lncRNAs can be directly regulated by miRNAs.^22^ It is unclear that whether the conversion of T > C in the rs3741219 polymorphism in the H19 could affect the miRNA-lncRNA interactions in breast cancer cells, and further studies are needed to explore the specific mechanisms.

H19 is an estrogen-regulated long noncoding RNA^29^ and harbors the microRNA-675 in its first exon. Previous investigations have indicated that the controlled release of miR-675 from H19 could inhibit estrogen-induced proliferation of ERa+ breast cancer cells.^30^ Compared with ERa-breast tumors, ERa+ breast tumors exhibited a higher expression of H19.^31^ In our study, we investigated the association between the H19 polymorphism and estrogen receptor (ER), progesterone receptor (PR), and human epidermal growth factor receptor-2 (HER-2) for the first time. In the case only analysis, rs217727CT genotype were associated with ER positivity (OR = 2.19; 95 % CI = 1.07–4.45) and HER-2 positivity (OR = 1.34; 95 % CI = 1.05–2.12), which was a novel finding. It is possible to propose the hypothesis that H19 genetic mutation may have interaction with ER and HER-2 during human breast tumorigenesis. It is worthwhile to better understand and clarify the underlying molecular mechanisms in future studies.

Haplotype was accepted as a group of correlated SNPs that are located in the same homologous chromosome and passed on to descendants together as a whole.^32^ Haplotype contained multiple SNPs information, and the statistical power of haplotype analysis was stronger than analysis of single SNP.^33^ This study analyzed haplotypes of rs3741219 and rs217727, and no significant positive or negative association with BC risk was found in the 4 haplotypes. This may because the borderline significant increased BC risk of rs3741219 could exhibit its restraint upon the significant reduced BC risk of rs217727. In order to find more credible evidence on the association between H19 haplotype and BC risk, further studies on larger populations are required.

The results of molecular epidemiology studies were always accompanied by high probability of false positive.^34–36^ In order to test the false-positive associations, we subsequently calculated the FPRP for all significant genetic effects observed in our study. The association between rs217727 CT+TT genotypes in subjects with no. of pregnancy >2 and breast cancer, and the positive association between individuals with 1 mutation allele and breast cancer were still noteworthy, which imply the functional SNPs in H19 might be involved in the breast cancer development with a high likelihood.

There were some strengths of this study that should be noted. First, although the role of H19 in multiple diseases has been reported, this is the first study investigating genetic variation of H19 in relation to breast cancer susceptibility. Second, the whole controls in our study were selected from people in a large sampling survey based on community, not from hospital, diminishing the effect of selection bias. To avoid the prevalence–incidence bias, the cases in our study were all newly pathological diagnosed. The controls and the cases were matched on age, and the baseline characteristic distributions were similar between the 2 groups. Therefore, we believed that selection bias was not substantial and not likely to influence the analyses of our study. Furthermore, for all significant genetic effects observed in our study, we further adjusted potential confounding factors in final analyses and calculated the FPRP. It is proved that our results were less likely to be false positives or false negative according to the FPRP results. However, some limitations of the present study should be mentioned. The statistical power of the present study may be restricted by the sample size and large proportion of missing data for some variable. And our study did not include detection of the influence of the H19 variants on its expression and activity. Therefore, further studies are worthwhile to validate whether H19 SNP could affect the expression of H19 and the etiology of BC.

In conclusion, the present population-based case–control study provided the first evidence that H19 rs217727 might play an interaction with ER positive and HER-2 positivity during human breast tumorigenesis. H19 genetic polymorphisms (rs3741219 and rs217727) may have no association with breast cancer risk after adjusted for reproductive variables. The study may require confirmation in larger prospective studies and different ethnicities with a gene expression functional assay.
